# Draft Genome Sequence of the Live Vaccine Strain *Brucella abortus* 82

**DOI:** 10.1128/genomeA.01101-13

**Published:** 2013-12-26

**Authors:** Alexander Shevtsov, Pavel Tarlykov, Elena Zholdybayeva, Elena Shevtsova, Dauren Momynkulov, Igor Sytnik, Talgat Karibaev, Andrey Chsherbakov, Kuvat Momynaliev

**Affiliations:** National Center for Biotechnology, Astana, Kazakhstana; National Veterinary Reference Center, Astana, Kazakhstanb

## Abstract

Vaccination is a crucial part of the brucellosis eradication programs worldwide. A live vaccine strain of *Brucella abortus* 82 has been successfully used for the vaccination of cattle against brucellosis in the former Soviet republics for the last 39 years. Here, we report the genome sequence of *Brucella abortus* 82.

## GENOME ANNOUNCEMENT

Brucellosis remains a major public health concern worldwide and is the most common zoonotic infection (1). The brucellosis eradication programs are based on the monitoring of epizootic processes, vaccination with the recommended live vaccines, or elimination of immunopositive animals (2). Since 1974, the former Soviet republics have officially started to use the live vaccine strain of *Brucella abortus* 82. As a result, the total number of infected farms in Russia fell by 80 times during the period of the successful application of the vaccine. In 2008, less than 70 farms were officially registered as infected (3). Success of this strain in vaccination is explained by the combination of a weak agglutination test response with high immunogenicity and comparatively high efficacy against brucellosis. In addition, strain 82 has low residual virulence and is stable during *in vitro* passaging in media and *in vivo* passaging in guinea pigs and cattle, including pregnant cows (3–5). Due to prolonged intracellular persistence of *Brucella* in the infected lymphoid tissues and probability of abortion in the vaccinated animals, genetic identification of *Brucella* isolates is required (6). Here, we report the draft genome sequence of *Brucella abortus* strain 82, which may help to elucidate the causes of low residual virulence and to develop a diagnostic approach for the differentiation of vaccine and infectious strains.

The genomic DNA was isolated from the strain *Brucella abortus* 82, deposited at the National Veterinary Reference Center in Astana, Kazakhstan. The whole-genome shotgun (WGS) sequence data were retrieved with the Ion Torrent sequencing platform, which gave 85-fold coverage. A total of 66 contigs with an *N*_50_ length of 107,330 bp were assembled using Newbler software v. 2.3 (454 Life Science). The genome was annotated using the NCBI Prokaryotic Genome Annotation Pipeline 2013. The draft whole-genome sequence of the strain *Brucella abortus* 82 has 3,250,206 bases that include 2,880 predicted coding sequences (CDS), 3 rRNA and 49 tRNA genes, and a GC content of 57.2%. Comparison of the *Brucella abortus* 82 genome with those of other vaccine or pathogenic strains may show genetic differences responsible for its phenotypic characteristics and pathogenicity. Further analysis and experimental confirmation will be described in our future work.

### Nucleotide sequence accession numbers.

This whole-genome shotgun project has been deposited at DDBJ/EMBL/GenBank under the accession no. AXBQ00000000. The version described in this paper is the first version, AXBQ01000000.
